# Time living with food insecurity and socio-demographic factors: longitudinal analysis in a city in the semi-arid region of Northeast Brazil

**DOI:** 10.1017/S1368980024000764

**Published:** 2024-03-27

**Authors:** Ana Beatriz Macêdo Venâncio dos Santos, Poliana de Araújo Palmeira, Angelo Giuseppe Roncalli da Costa Oliveira

**Affiliations:** 1 PhD Student of the Graduate Program in Public Health, Federal University of Rio Grande do Norte. Av. Sen. Salgado Filho, Lagoa Nova, Natal, RN, Brazil; 2 Professor of the Bachelor’s Degree in Nutrition, Federal University of Campina Grande, Cuité, PB, Brazil; 3 Professor of the Graduate Program in Public Health, Federal University of Rio Grande do Norte, Natal, RN, Brazil

**Keywords:** Food insecurity, Socio-demographic factors, Cohort studies, Brazil

## Abstract

**Objective:**

Food insecurity (FI) is the lack of daily access for everyone to quality food in sufficient quantity. In many populations, it presents as a chronic and persistent condition. This study analysed the association between the length of time living with FI and socio-demographic conditions in households in a semi-arid municipality in the Brazilian Northeast between 2011 and 2019.

**Design:**

This is a population-based cohort study among families in the municipality in Northeast Brazil (2011, 2014 and 2019). FI was estimated through the Escala Brasileira de Insegurança Alimentar (EBIA, Brazilian Household Food Insecurity Measurement Scale), and the longitudinal category of time of living with FI was adopted to classify them according to the time they remained in FI during the cohort. The association with the socio-demographic profiles of the population was verified through multinomial logistic regression.

**Setting:**

Households in semi-arid, Northeast of Brazil.

**Participants:**

Household respondents interviewed in 2011, 2014 and 2019 (*n* 274).

**Results:**

Sixty-seven percentage (67 %) of families lived in FI in this period. Rural residence, low monthly per capita income and low schooling of the household reference person increased the chances of these families living longer in FI. These overlapping conditions increased the odds of FI in the household.

**Conclusions:**

Coping with FI requires intersectoral intervention that improves the socio-demographic conditions of the population.

Food security (FS) is physical, social, economic and continuous access to safe, nutritious and sufficient food to meet food needs and preferences that promote a healthy life^(1)^. Deprivation in access to food, that is, food insecurity (FI), is associated with adverse social conditions. In the Brazilian context, the FI is more prevalent among families in the North and Northeast regions, with lower family income, higher household density and among the female sex, black/brown colour and low schooling of the household person reference^(2–4)^.

The monitoring of the FS situation in the household is carried out internationally through the Food Insecurity Experience Scale (FIES) and, in Brazil, the Brazilian Household Food Insecurity Measurement Scale (Escala Brasileira de Insegurança Alimentar – EBIA) is adopted, adapted to the Brazilian context and recognised by researchers and the federal government of Brazil^(5,6)^. Lignani and collaborators^(2)^, given the association of FI with the social conditions of the population, highlight the scale as a broad and consistent index of social vulnerability.

However, despite researchers discussing the relationship between socio-demographic conditions and FI in Brazil^(3,4)^, including in the semi-arid Northeast^(6)^, most of these studies are cross-sectional and, therefore, have limitations regarding the possibility of understanding this relationship over time in the same families.

Given the gap, a longitudinal cohort analysis was applied to answer the question of this study: When adding the time factor to the analysis, which socio-demographic characteristics are associated with FI in the household among families in the Brazilian semi-arid region? In this sense, this article aims to analyse the association between socio-demographic conditions and the time of living in FI in the households of a municipality located in the semi-arid region of Northeast Brazil between 2011 and 2019.

## Methodology

### Type of study, scenario, sample and data collection

**Fig. 1:**
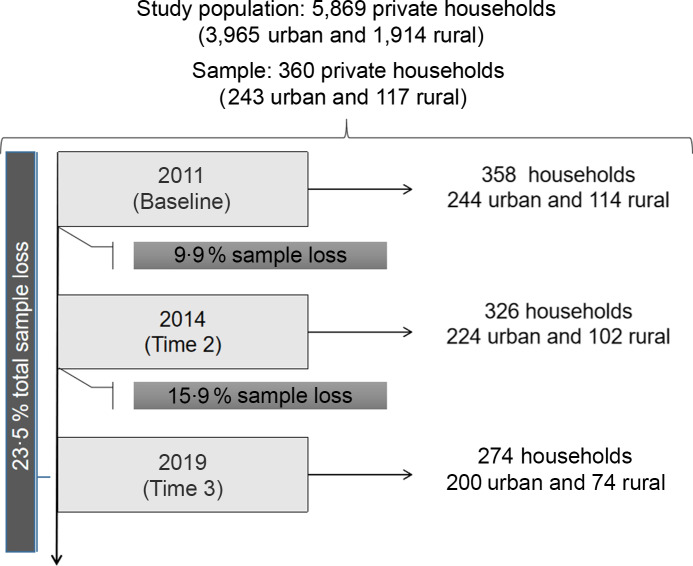
Flowchart of the research sample, highlighting the whole sample used in this study, 2023

A population-based longitudinal cohort study was conducted in the city of Cuité, Paraíba, Northeast Brazil. Located 273 km from the capital of Paraíba, it has approximately 20 000 inhabitants, a territorial extension of 733·82 km^2^, and a low Human Development Index (0·59)^(7)^. We performed this cohort study in three follow-up periods: 2011 (baseline–time 1), 2014 (time 2) and 2019 (time 3) (Fig. 1).

The baseline research was performed from a random and stratified sampling of 5.896 households registered in the municipality (*n* 360 households), with urban (*n* 243) and rural (*n* 117) representativeness, considering a 95 % CI and 5 % sampling error. Further details on the cohort baseline are available in Palmeira, Salles-Costa and Pérez-Escamilla^(8)^. Cohort stages were carried out in 2014 and 2019 to monitor changes in FI considering the implementation of the Brazil Without Poverty Strategy (2011) and the impacts of the crisis established in Brazil with the impeachment of President Dilma Rousseff and the adoption of fiscal austerity measures (2016), respectively.

Data collection was carried out by previously trained students undergraduates of the Nutrition Course at the Federal University of Campina Grande in the following periods: in time 1 – between May and June 2011 (358 households); in time 2 – between May and September 2014 (326 households); and in time 3 – between August and December 2019 (274 households). During the collection, supervisors performed data quality control and reviewed the questionnaires after application to assess the information’s consistency and identify missing data. We returned to the families’ homes when necessary to verify and confirm information.

In times 2 and 3 (2014 and 2019), after returning to the households surveyed in time 1, a sample loss of 9·9 % and 15·9 % was obtained, respectively. Thus, the sample of this study is composed of 274 families followed in the three follow-ups, resulting in a sample attrition of 23·5 %. This sample size can detect a true OR of at least 3·54 with a confidence of 95 % and 80 % power (1-beta), assuming an occurrence of the result among those not exposed. The randomness of the missing data was tested using Little’s MCAR Test between the independent variables and the outcome, described below.

### Outcome variable

The 14-item version of the EBIA was used to measure FI. The EBIA is an experience-based scale adapted from the US Household Food Security Survey Module validated in Brazil since 2003. Based on its theoretical underpinnings, this instrument considers FI to be a progressive phenomenon experienced by households on severity levels^(5)^. We classified the households according to the sum of affirmative responses to EBIA items and household composition: (i) ‘food security (FS)’ (score = 0); (ii) ‘mild’ FI (score = 1–5 in households with children/adolescents, 1–3 in adult-only households); (iii) ‘moderate’ FI (score = 6–9 in households with children/adolescents, 4–5 in adult-only households) and (iv) ‘severe’ FI (score = 10–14 in households with children/adolescents, 6–8 in adult-only households).

We assessed the length of time living with FI over time based on four longitudinal categories: (1) persistent food security (persistent FS) when the family was in FS over time; (2) FI at one time (FI-1) when the family lived in FI in at least one of the times of the cohort, regardless when it was; (3) FI at two times (FI-2), when the family lived in FI in two of the times of the cohort, regardless of when it was and (4) persistent food insecurity (persistent FI), when the family lived in FI in the three times of the cohort.

### Predictor variables

The independent variables used were housing area (rural/urban), number of residents in the household (mean), age (mean), sex (female/male), education level (low schooling: up to elementary school/high schooling: high school or more), occupation of the household reference person (retired or pensioner/paid occupation/unpaid occupation) and monthly family income per capita (above minimum wage/below minimum wage). During the interview, the household reference person was identified as responsible for the family. The Brazilian minimum wage was in 2011, R$ 545 ($US 340·6); in 2014, R$ 724 ($US 308·1) and in 2019, R$ 998 ($US 253·3).

The longitudinal variable categories described below were defined to prospectively identify the dynamics of change in the conditions analysed over time. With this, we observed the change of category during the cohort. We grouped families that remained in the same category over time and migrated to this category, maintaining their classification until the end of the cohort (2019):

1. Housing area: urban over time or changed from rural to urban area; rural over time or changed from urban to rural area;
2. Monthly family income per capita: Above minimum wage over time or changed to this classification; below minimum wage over time or changed to this classification;
3. Sex of household reference person: male sex over time or changed from female to male; female sex over time or changed from male to female;
4. Schooling of household reference person: high schooling over time or changed from low to high schooling; low schooling over time or changed from high to low schooling; and
5. Occupation of household reference person: retired/pensioner over time or changed to this classification; paid occupation over time or changed to this classification and unpaid occupation over time or changed to this classification.

Considering the unit of analysis in this study is the family, different people may have played the role of the household reference person over time. The family asked about the positions of each family member within the household, including the reference person. Thus, the longitudinal categories of predictor variables, including sex and colour, can show changes and variations over time.

### Data analysis and longitudinal categories

In the bivariate analysis, we estimated the prevalence of FI and socio-demographic variables in 2011, 2014 and 2019, and to evaluate the changes over time, we applied Cochran’s *Q* tests (categorical variables) and ANOVA (quantitative variables). We applied Pearson’s chi-square test to analyse the association between time living with FI and longitudinal predictive variables. We also used the multinomial logistic regression with robust variance adjustment to estimate the association between time living with FI and predictive variables with a significance level of up to 20 % in the previous stage (*P* < 0·20).

The model included housing area, income, and education level and adjusting variables (number of residents in the household, age and occupation of the household reference person). We estimate the probabilities of families with different vulnerability profiles (predicted variables) living with FI over time and illustrate the results using the margin command. All models were tested for collinearity, and the accuracy (78·6 %) was analysed. The analyses were developed using Stata Software 15.0, with 95 % CI and 5 % significance level.

## Results

### Characteristics of the cohort families

Table 1 describes the characteristics of the population studied over time. At baseline, a majority of urban population (71·2 %) was observed, with income below Brazilian minimum wage (62·4 %), an average of 3·6 residents per household and household reference person, female (55·1 %), black, brown or other (60·2 %), with low schooling (80·2 %), paid occupation (50·2 %) and average age of 50 years. Regarding the situation of FS in the household, 52·5 % were in FI, with 30·9 % mild, 12·4 % moderate and 9·8 % severe.

**Table 1:** Socio-demographic characteristics of the population in Cuité, Northeast, Brazil, in 2011, 2014 and 2019 (*n* 274)

Socio-demographic variables	2011	2014	2019	*P*
	(%)	(%)	(%)	
**Household**				
Area of residence				
Urban	71·2	71·5	73	0·223†
Rural	28·8	28·5	27	
Monthly family per capita income				
Above 1 minimum wage	37·6	51·1	49·6	<0·001†,*
Below 1 minimum wage	62·4	48·9	50·4	
Household density				
Mean number of residents in the household	3·6	3·6	3·3	<0·001‡,*
Food security and insecurity levels				<0·001†,*
Food security	47·5	62	65·2	
Mild food insecurity	30·3	21·9	18·7	
Moderate food insecurity	12·4	8·4	10·2	
Severe food insecurity	9·8	7·6	5·9	
**Household reference person** §				
Sex				
Male	44·9	44	43·8	0·885†
Female	55·1	56	56·2	
Age				
Mean age	50·3	52·9	57·1	<0·001‡,*
Skin colour/ethnicity				
White	39·8	38·0	34·3	0·059†
Black/brown/other	60·2	62·0	65·7	
Education				
High schooling (high school or more)	20·2	21·9	21·6	0·527†
Low schooling (up to elementary school)	79·8	78·1	78·4	
Occupation				
Retired or pensioner	32·6	37·6	34·3	0·192†
Paid occupation	50·2	50·2	50	
Unpaid occupation	17·2	12·2	15·7	

Value of the minimum wage in the years of collection: R$ 545·00 = US$ 305·66 (2011), R$ 724·00 = US$ 279·66 (2014) and R$ 998·00 = US$ 236·85 (2019).

* *P* < 0·05.

† *Q* Cochran test.

‡ ANOVA test.

§ Person identified as responsible for the family. Depending on the family composition, this person changed during the cohort time.

Times 2 (2014) and 3 (2019) of the cohort showed statistically significant differences between the prevalence of the three times regarding family income (*P* < 0·001), the situation of FS in the household (*P* < 0·001), mean number of residents (*P* < 0·001) and mean age of the household reference person (<0·001). Regarding income, there was an increase in the family income mean in 2014 by US$ 202,41 (R$ 524·00 Brazilian real) and a reduction in 2019 by US$ 99,55 (R$ 419·50 Brazilian real), as well as the prevalence of families with income more than one minimum wage increased and reduction in 2014 and 2019, respectively. In the FS situation, there was a considerable reduction of families in FI between time 1 and time 2 for all severities. Between times 2 and 3, there was a slowdown of this reduction and an increase of moderate FI.

### Persistent food security associated with predictor variables

Our findings revealed 34·3 % of households in persistent FS and 65·7 % of families who lived with FI over time, with 23·3 % living in FI at one time (FI-1), 24·1 % in two moments (FI-2) and 18·3 % over time (persistent FI). Table 2 shows the change in socio-demographic characteristics throughout the cohort according to the groups living in FI at different times (Table 2). The results show a scenario of greater vulnerability among families living in FI, especially persistent FI. The area of residence, family income and education levels of the household reference person were statistically associated with living in FI (*P* < 0·001). The relationship observed is that the longer the time living in FI, the higher the prevalence of classification of families in a risk condition (rural, family income below minimum wage and low schooling).

**Table 2:** Changes in socio-demographic characteristics throughout the cohort, according to time living with food insecurity in the household, Cuité, Northeast, Brazil, 2011–2019

Variables with longitudinal categories	Persistent food security (*n* 94)	Insecurity at one time in cohort (*n* 64)	Insecurity at two times in cohort (*n* 66)	Persistent food insecurity (*n* 50)	*P*
	(%)	(%)	(%)	(%)	
**Household throughout the cohort**					
Area of residence					<000·1*
Urban over time (or changed)	87·2	78·1	68·9	48	
Rural over time (or changed)	12·7	21·9	31·8	52	
Monthly family per capita income					
Above minimum wage over time (or changed)	76·6	67·2	39·4	18	<000·1*
Below minimum wage over time (or changed)	23·4	32·8	60·6	82	
**Household reference person throughout the cohort** †					
Sex					0·805
Male over time (or changed)	43·6	48·4	47	40	
Female over time (or changed)	56·3	51·6	53	60	
Skin colour/ethnicity					0·443
White over time (or changed)	39·4	28·1	36·4	30	
Black/brown/other over time (or changed)	60·6	71·8	63·6	70	
Education					<000·1*
High schooling over time (or changed)	42·2	19·1	6·3	8·7	
Low schooling over time (or changed)	57·8	80·9	93·7	91·3	
Occupation					0·341
Retired/pensioner over time (or changed)	42·5	33·3	40·0	22·4	
Paid occupation over time (or has changed)	44·7	50·8	46·1	63·3	
Unpaid occupation over time (or changed)	12·8	15·9	13·9	14·3	

* The Pearson’s chi-square test indicates association was statistically significant (*P* < 0·05).

† Person identified as responsible for the family. This person can changed during the cohort time. Housing area: urban over time or changed from rural to urban area; rural over time or changed from urban to rural area; monthly family income per capita: above minimum wage over time or changed to this classification; below minimum wage over time or changed to this classification – value of the minimum wage: R$ 545·00 (2011), R$ 724·00 (2014) and R$ 998·00 (2019); sex: male sex over time or changed from female to male; female sex over time or changed from male to female; schooling: high schooling over time or changed from low to high schooling; low schooling over time or changed from high to low schooling; and occupation: retired/pensioner over time or changed to this classification; paid occupation over time or changed to this classification; and unpaid occupation over time or changed to this classification.

Table 3 presents the results obtained in the multinomial logistic regression and points out that a rural household over time (or changed) has a higher chance of presenting persistent FI when compared with families in persistent FS, as well as families with an income below one minimum wage over time (or changed) of living with FI at two moments and persistent FI. In the case of low schooling of the household reference person over time (or changed), there is a higher chance of residing in FI in any of the classifications (FI-1, FI-2 and persistent FI).

**Table 3:** OR of time living with food insecurity according to socio-demographic characteristics among families in the municipality of Cuité, Northeast, Brazil, 2011–2019

Socio-demographic characteristics	Families in food insecurity at one time of the study (*n* 64)				Families in food insecurity at two times of the study (*n* 66)				Families in persistent food insecurity (*n* 50)			
	OR	95 % CI	Adjusted† OR	95 % CI	OR	95 % CI	Adjusted† OR	95 % CI	OR	95 % CI	Adjusted† OR	95 % CI
Rural household over time (or changed)	1·91	0·81, 4·47	1·40	0·59, 3·32	3·18*	1·43, 7·08	2·19	0·89, 5·35	7·40*	3·25, 16·8	4·86*	1·76, 13·4
Below minimum wage over time (or changed)	1·59	0·78, 3·24	1·36	0·57, 3·26	5·03*	2·53, 10·0	3·24*	1·44, 7·29	14·9*	6·26, 35·5	6·52*	2·20, 19·3
Low schooling over time (or changed)	2·94*	1·38, 6·23	3·25*	1·44, 7·30	10·5*	3·52, 31·4	9·70*	2·94, 31·9	7·46*	2·47, 22·5	5·23*	1·71, 16·0

Housing area: urban over time or changed from rural to urban area; rural over time or changed from rural to urban area; monthly family income per capita: above minimum wage over time or changed to this classification; below minimum wage over time or changed to this classification – value of the minimum wage: R$ 545·00 (2011), R$ 724·00 (2014) and R$ 998·00 (2019); schooling: high schooling over time or changed from low to high schooling; low schooling over time or changed from high to low schooling.

* *P* < 0·05.

† Multinomial logistic regression with model including variables with up to 20 % of significance and adjusted for longitudinal occupation of household reference person, mean number of residents and mean age in the three times.

**Fig. 2:**
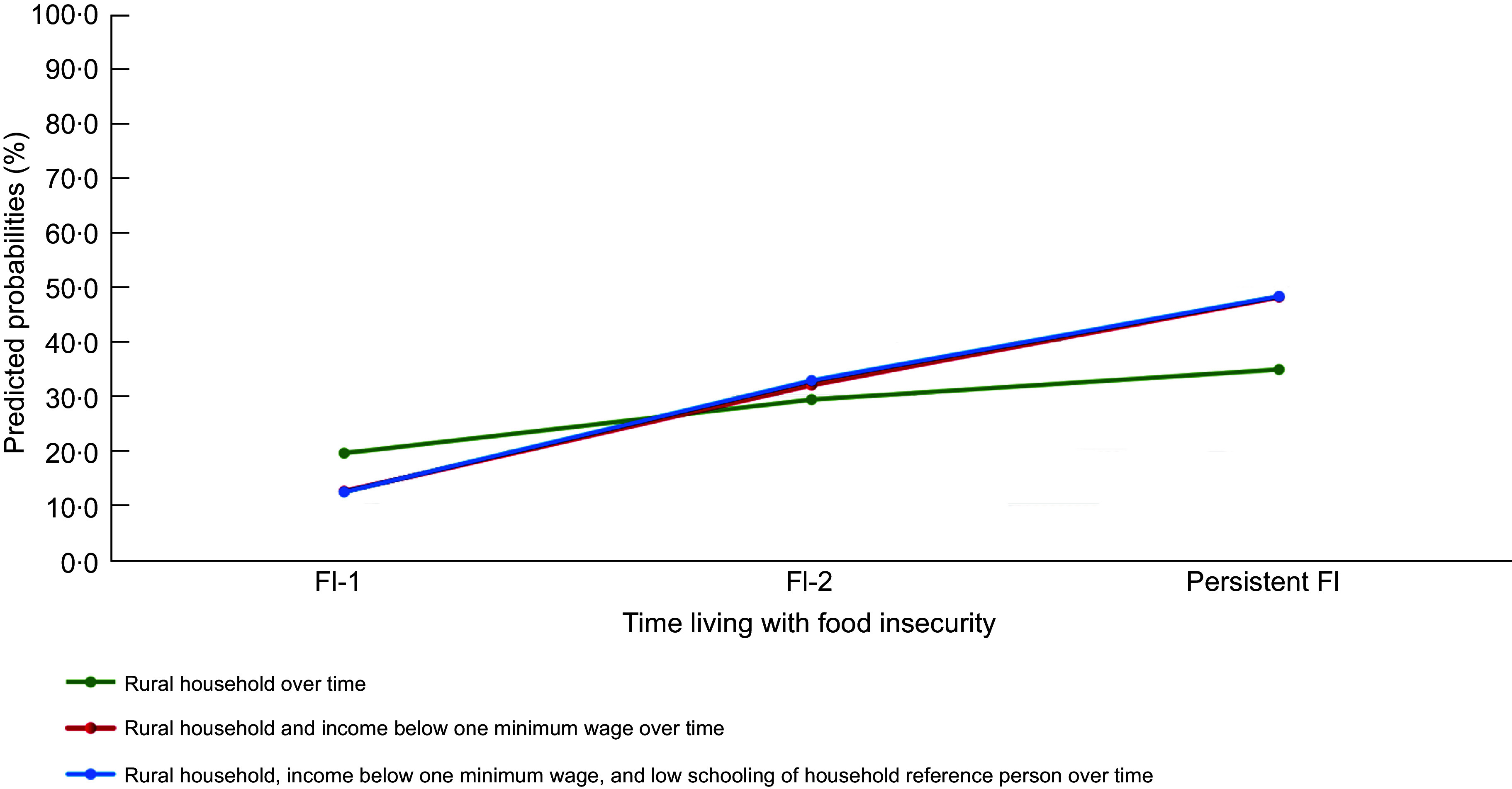
Predicted probabilities for the different categories of time living in food insecurity, according to vulnerability profiles throughout the cohort, Cuité, Northeast, Brazil, 2011–2019

Figure 2 shows the predicted probabilities for the outcomes of living in FI-1, FI-2 and persistent FI, according to the vulnerability profiles of families across the cohort: rural families (profile 1), rural families with low incomes (profile 2) and low-income rural families and a household reference person with low schooling (profile 3). The odds gradually increase as time with FI increases for all family’s profiles. For families with rural residence (exclusively), the probabilities increase when looking at FI-1 (19·4 %) and persistent FI (34·7 %). This increase is more expressive for households that overlap the three risk conditions (FI-1: 12·3 %; persistent FI: 48·1 %).

## Discussion

The objective of this article was to analyse the time living with FI during the cohort and the associated socio-demographic factors. The prevalence of FI in the studied households decreased over time, although 65·7 % of the families had experienced FI during the cohort. Regarding changes in socio-demographic conditions, the results revealed a reduction in the average number of residents in the household and instability in monthly family income per capita, which increased in 2014 and decreased in 2019. Other characteristics remained similar to what was observed in the study baseline.

Our findings showed that the change in the situation of FS and FI severities between 2011 and 2014 is similar to that verified for the Brazilian population by the Penssan Network^(3)^, through EBIA applied in other national studies^(9–11)^. However, they present differences in the 2014–2019 scenario, considering that there was a worsening of FI for all severity at the national level, while in the families studied, there was an increase in the prevalence of moderate FI. Latin America, through FIES, also showed a worsening of the FS scenario, where there was a reduction in the prevalence of FS between 2014 (51 %) and 2017 (43 %), with an increase in moderate and severe FI. Argentina, Ecuador and Brazil showed a more pronounced decline due to the worsening of the economic context of these countries in the same period, reaching a 24 % reduction in FS in Brazil^(12)^.

Although the results of this article are better for the FS situation than those observed nationally and internationally when considering the prevalence of cross-sectional collections, the longitudinal analysis of the time living with FI in the household in the present study elucidates the alarming data of 65·7 % of families living in FI between 2011 and 2019 in the municipality.

Palmeira *et al.* ^(8)^, based on times 1 and 2 of this same research, observed that only 37·4 % of households were safe at both times (2011 and 2014) and described the following dynamics of change in the FI situation for families insecure at baseline: households that remained in FI (29·8 %), that moved from FI to FS (24·5 %) and that moved from FS to FI (8·3 %). After an increase of 5 years of monitoring families, we observed the continued high prevalence of FI and a reduction in the prevalence of households in persistent FS when compared with the study carried out with the first follow-up (2011 and 2014), and this article considered in the three periods (2011, 2014 and 2019), obtaining 37·4 % and 34·7 % of families in permanent FI, respectively.

There is, therefore, the permanence of families in FI for an extended period (24·1 % in FI-2 and 18·3 % in persistent FI) and the instability in FS at households for families who lived in FI-1 (23·3 %). The persistence of FI was also observed in other municipalities in Paraíba, northeastern Brazil, in 48·9 % of the families studied with moderate/severe FI in 2005 and 2011, according to Cabral *et al.* ^(13)^.

In a study carried out with households in South Carolina (USA) between 2013 and 2016, the authors identified FI as a chronic condition in 37 % of the despite improvements in the territory’s economy^(14)^. Also, in the USA, between 1995 and 2015, the persistent FI was 4·9 % among families in Michigan, USA (2011–2015)^(15)^ and 21 % in the USA (1995–2015)^(16)^. It is noteworthy that stability is one of the dimensions of the FS and can be seen as a premise for its guarantee, given the importance of continuing the availability, accessibility and use of nutritious foods at all ages appropriate to each life cycle^(1,17)^.

The factors statistically associated with a more extended time living with FI were the area of residence, income and schooling of the reference person in the household. Living in rural areas, having a family income of less than the minimum wage and having a household reference person with low schooling in the entire cohort (or who moved to one of these categories) were risk conditions.

FI also showed a higher prevalence among families living in rural areas in studies in the Brazilian semi-arid^(18)^, the central-west region^(19)^ and the national territory^(4)^. The vulnerability of rural territories is recognised by the literature that cites different reasons, such as the greater concentration of poverty and the difficulty in accessing food, goods, services, transport^(20)^ and health services^(21)^. Specifically in the semi-arid region, there is difficulty accessing water, in quantity and quality, for the different daily tasks (domestic use, animal husbandry and planting)^(22)^. The water insecurity of the region worsened between 2012 and 2018 because of the multiannual drought that lowered the water levels of the reservoirs in this region^(23)^.

The guarantee of the FS in the semi-arid region requires a better coexistence with the drought and, therefore, the implementation and strengthening of public policies that enable a dignified life for the population^(22)^, such as the ‘One Million Cisterns Program’ that suffered from the dismantling of public policies in Brazil from 2015^(24)^.

Concerning income and education, the recent national and international literature^(17,19)^ discussed that low income and low schooling of the household reference person as determinants of FI increase the chance of FI in the family. The debate also includes the inequality with which political and economic crises have affected the population, given its effects on income and educational levels^(2)^.

There is a robust discussion about the direct association between income and the outcome of FI in Brazil^(2,3,25)^. The results of the longitudinal study carried out by Palmeira *et al.* ^(8)^, which analysed the effects of income and the increase in the Bolsa Família Program resource on the FI of families, reinforce the relevance of direct transfer programmes since if Bolsa Família did not exist, approximately 10 % of families that left the FI condition between 2011 and 2014 would have remained unsafe.

Low schooling was associated with all the outcomes of time living with FI (FI-1, FI-2 and persistent FI). The result endorses the importance of education in guaranteeing the FS as an instrument for overcoming the cycle of poverty of families^(2,19)^. Lower schooling can affect the family’s financial management and the maintenance of adequate nutrition and make accessing professional opportunities with better conditions difficult.

Education is a constitutional right in Brazil^(26)^, and the government must guarantee quality public education for all. Considering the low access to schools and universities and scholar dropout rates, over the years, the Brazilian government has implemented initiatives to expand access to education, promote student assistance (housing, transport, food and student scholarships) and improve the infrastructure of the school environment and the quality of teaching^(27)^. The federal investments to fund education grew between 2005 and 2014. However, after 2015, a heavy political and economic crisis started, resulting in the implementation of a fiscal austerity project that reduced education investments to amounts similar to those of 2002^(28)^.

During our research, the Brazilian score position on the Human Development Index, which includes education, fluctuated from 84th in 2011^(29)^ to 79th in 2014^(30)^, backing to 84th in 2019^(31)^, showing how public investments are necessary. Social inequality determines the schooling rates in Brazil. Because of this, if social conditions affect school performance, in addition to educational policies, it is urgent to implement a set of policies to reduce inequities that historically excluded populations such as women, black people and rural families from education opportunities^(32)^.

In addition to scoring the risks of living in rural areas, having low family income and household reference persons with low schooling, this study reveals the increased probability of living in FI for longer in the face of overlapping vulnerabilities of the aggregate family. Considering the long time living with FI during the last eight years, there is a stagnation in the living conditions of these families in the northeastern semi-arid region, which, according to Campello *et al.* ^(17)^, is part of the hard core of poverty that remained in poverty. Despite federal investments to fight poverty and the process of inclusion of the most vulnerable experienced in the country during the Lula government (2003–2010), extreme poverty is still a reality in this region of Brazil.

In the Brazilian scenario, these studies can contribute to better-guiding actions that integrate the axes of action of federal, state and municipal governments in light of the Brazil Without Hunger Plan (2023), launched with the objective of overcoming hunger in the country by 2030, the reduction of FI at all levels and poverty rates. This study, despite having been carried out with a specific population from one of the 1,262 municipalities in the Brazilian semi-arid region, denounces the problem of the persistence of FI, given the persistence of precarious living conditions over time. Furthermore, approximately 70 % of Brazilian municipalities have less than 20 thousand inhabitants, that is, poor municipalities with low political-administrative capacity, whose challenges to implementing local and national policies are enormous.

The maintenance of social inequalities already observed in Brazil and several semi-arid areas and countries are cyclically reinforced when people do not have equal opportunities to have a dignified life with guaranteed rights to food, health, education, income, housing and work and continue to be marginalised by society and neglected by development policies. Therefore, the adoption of a development project that considers implementing and strengthening public policies aimed at reducing social inequalities is defended, especially about income generation, valorisation of the minimum wage, expansion of universal access to quality education and better coexistence with the semi-arid and living conditions for the rural population.

A limitation of this study was the sample size and attrition over time, which could minimise the accuracy of the statistical analysis. However, the longitudinal dataset with three follow-ups allowed the development of robust analysis and the debate on FI persistence. Moreover, we evaluated the dataset’s quality based on randomness and power criteria. Another area for improvement was the impossibility of verifying families’ changes between the four FS or FI levels throughout the cohort due to the sample size and the complexity of the changes over time. However, the variable time living with FI is an innovative approach to analysing the persistence of FI that could be reproduced and applied in future studies.

### Conclusions

Our findings revealed the high prevalence of families living in FI for prolonged periods and the possibility of FI staying in the household, given the maintenance of the vulnerable conditions of the population. Understanding and diagnosing chronic and persistent hunger are essential to direct and encourage structuring actions to achieve sustainable development goals and eliminate hunger, especially when there is a global search for strategies to overcome hunger after the accentuation of economic and social crises.

It is suggested that new national and international studies be carried out that consider the time factor in evaluating FI and its determining factors, as well as qualitative studies that identify themselves as survival strategies for families living with FI. In addition to population studies, it is recommended that studies be carried out that analyse the formulation of structuring public policies and their impacts on breaking the cycle of vulnerabilities that contribute to the persistent condition of FI.

## Supporting information

Santos et al. supplementary material Santos et al. supplementary material

## Supplementary material

For supplementary material accompanying this paper visit https://doi.org/10.1017/S1368980024000764
