# Supplementary material for: Time living with food insecurity and socio-demographic factors: longitudinal analysis in a city in the semi-arid region of Northeast Brazil
Source: Public Health Nutr. 2024 Mar 27;27(1):e108. doi: 10.1017/S1368980024000764 (PMC11036425; doi:10.1017/S1368980024000764)
Supplement: Santos et al. supplementary material [file S1368980024000764sup001.docx]

| Supplementary Table 1: Changes in sociodemographic characteristics throughout the cohort in the household, Cuité, Northeast, Brazil, 2011-2019**.** | | |
| --- | --- | --- |
| **Variables with longitudinal categories** | **%** | **n** |
| **Households throughout the cohort** |  |  |
| Area of residence |  |  |
| Urban over time | 69.7 | 101 |
| Changed to urban area | 3.6 | 10 |
| Rural over time | 25.2 | 69 |
| Changed to a rural area | 1.5 | 4 |
| Monthly Family Income per Capita |  |  |
| Above minimum wage over time | 27.4 | 75 |
| Changed to above minimum wage | 27.4 | 75 |
| Below minimum wage over time | 37.6 | 103 |
| Changed to below minimum wage | 7.7 | 21 |
| **Household reference person** |  |  |
| Sex |  |  |
| Male over time | 38.7 | 106 |
| Changed to male | 6.2 | 17 |
| Female over time | 49.3 | 135 |
| Changed to female | 5.8 | 16 |
| Skin color /Ethnicity |  |  |
| White over time | 25.2 | 69 |
| Changed to white color | 9.1 | 25 |
| Black/brown/other over time | 52.2 | 143 |
| Changed to black/brown/other colors | 13.5 | 37 |
| Education |  |  |
| High schooling over time | 16.5 | 45 |
| Changed to high schooling | 5.5 | 15 |
| Low schooling over time | 74.3 | 202 |
| Changed to low schooling | 3.7 | 10 |
| Occupation |  |  |
| Retired/pensioner over time | 22.7 | 62 |
| Changed to retired/pensioner | 13.9 | 38 |
| Paid occupation over time | 34.1 | 93 |
| Changed to a paid occupation | 15,4 | 42 |
| Unpaid occupation over time | 4.4 | 12 |
| Changed to unpaid occupation | 9.5 | 26 |
| ^value^ of the minimum wage in the years of collection: R$ 545.00 (2011). R$ 724.00 (2014) and R$ 998.00 (2019). | | |
|  | | |
